# Integrative Metabolomic and Metallomic Analysis in a Case–Control Cohort With Parkinson’s Disease

**DOI:** 10.3389/fnagi.2019.00331

**Published:** 2019-12-06

**Authors:** Marianna Lucio, Desiree Willkommen, Michael Schroeter, Ali Sigaroudi, Philippe Schmitt-Kopplin, Bernhard Michalke

**Affiliations:** ^1^Analytische BioGeoChemie, Helmholtz Zentrum München, Neuherberg, Germany; ^2^ Klinik und Poliklinik für Neurologie, Uniklinik Köln, Cologne, Germany; ^3^Klinik für Klinische Pharmakologie und Toxikologie, Universitätsspital Zürich, Zurich, Switzerland; ^4^Institut I für Pharmakologie, Zentrum für Pharmakologie, Uniklinik Köln, Cologne, Germany; ^5^Chair of Analytical Food Chemistry, Life Science Center Weihenstephan, Technical University of Munich, Munich, Germany

**Keywords:** data integration, metabolomics, metallomics, Block-sPLS-DA, Parkinson’s disease

## Abstract

Parkinson’s disease (PD) is a neurodegenerative disease with a complex etiology. Several factors are known to contribute to the disease onset and its progression. However, the complete underlying mechanisms are still escaping our understanding. To evaluate possible correlations between metabolites and metallomic data, in this research, we combined a control study measured using two different platforms. For the different data sources, we applied a Block Sparse Partial Least Square Discriminant Analysis (Block-sPLS-DA) model that allows for proving their relation, which in turn uncovers alternative influencing factors that remain hidden otherwise. We found two groups of variables that trace a strong relationship between metallomic and metabolomic parameters for disease development. The results confirmed that the redox active metals iron (Fe) and copper (Cu) together with fatty acids are the major influencing factors for the PD. Additionally, the metabolic waste product p-cresol sulfate and the trace element nickel (Ni) showed up as potentially important factors in PD. In summary, the data integration of different types of measurements emphasized the results of both stand-alone measurements providing a new comprehensive set of information and interactions, on PD disease, between different variables sources.

## Introduction

Parkinson’s disease (PD) is a progressive neurodegenerative disease affecting mainly people older than 50 years (de Rijk et al., 1997). As the world population ages, the total amount of people affected by PD is increasing (Tanner and Goldman, 1996). Therefore, this disease will gain always more interest in research. Up to now, the underlying mechanisms are not fully understood. Several factors influencing the onset of PD have already been found, highlighting the multifactorial etiology of the disease. Environmental and occupational factors like exposure to metals (Witholt et al., 2000; Lucchini et al., 2007; van der Mark et al., 2015; Gunnarsson and Bodin, 2017), pesticides and fungicides (Uversky, 2004; Hancock et al., 2008) as well as genetic factors (Dawson, 2003; Gilks et al., 2005), aging (Sofic et al., 1988; Gaeta and Hider, 2009), and biological factors like aggregation and misfolding of proteins (Paik et al., 1999; Uversky et al., 2002) seem to be involved in the onset and in the progression of PD.

Various studies are discussing the involvement and changes of several metals in biofluids of PD patients in comparison to healthy controls (Jiménez-Jiménez et al., 1992; Forte et al., 2004; Zhao et al., 2013; Sanyal et al., 2016; Maass et al., 2018). Therefore, a great number of metals have been analyzed with varying results.

On the other hand, the metabolic changes through disease are discussed in literature. Differing biofluids showed altered concentration profiles within various compound classes. Compound classes like sugars, fatty acids, and AAs (Wishart et al., 2007; Okuzumi et al., 2019; Socha et al., 2019) or lipids (Xicoy et al., 2019) were found to be affected through disease.

These fields of either metallomics or metabolomics alone already show interesting results and changes and ongoing in diseased individuals. Even more interesting are the possible relations between these datasets within the same study population opening new perspectives and hypothesis on this degenerative disease. Therefore, hereby, we processed for the first time, to our knowledge, the data integration of these different types of measurements. The results may lead to even enhanced knowledge of potential causative changes associated with the PD status.

The aim of this work was to integrate different variables, determined by diverse methods of investigations, and related to the PD patients. More specifically, we reprocessed previous results from metabolomics and metallomics studies on the samples set, as published in Willkommen et al. (2018a, b), respectively, who have used the same set of 33 PD patients and 101 neurologically healthy controls. Therefore, the possible explored relations between different parameters can deliver additional new information on important biological processes in PD that are impossible to uncover otherwise. The statistical evaluations were built up between the “most important m/z signals” from ESI-FT-ICR-MS analysis and the metal concentration information and ratios information. The “most important m/z signals” refer to the list of annotated masses published in Willkommen et al. (2018a) and considered as the list of metabolites obtained after the feature selection algorithm (Supplementary Table S2). The metals considered in this study are zinc (Zn) and the redox-active metals like iron (Fe), copper (Cu), and manganese (Mn). Additionally, the metallomic parameters refer to the species characterization measurements and the amount of metals within AA fractions. The data were evaluated with a Block Sparse Partial Least Square Analysis (Block-sPLS). This analysis is suitable to retrieve the mutual relation between different types of datasets (in this case, metallomic–metabolomic data) with a specific focus on variables selection. Moreover, this technique offered several graphical outputs that enable better knowledge of the relationships and correlation structures between the data integrated.

## Materials and Methods

### Chemicals

The purchased chemicals were as follows: MeOH from CHROMASOLV^®^ LC-MS (Sigma-Aldrich, St. Louis, United States), L-arginine from Sigma-Aldrich (>98% purity, St. Louis, United States), Tris (Carl Roth, Karlsruhe, Germany), MeOH (Merck, Darmstadt, Germany), elemental standards (Perkin Elmer, Rodgau-Jügesheim, Germany), NH_4_Ac, human serum albumin (99%), bovine-γ-globulin, bovine-apo-transferrin (98%), dichloromanganese tetrahydrate (99.99%), citric acid, reduced and oxidized glutathione, arginase, ferritin, and β-lactoglobulin (each from Sigma-Aldrich, Steinheim, Germany).

### Study Participants

A total of 134 samples of cerebrospinal fluid (CSF) were taken by standardized lumbar puncture at the Cologne University Hospital (Germany). Even though originally not intended for scientific use, the samples were stored in the biobank of the hospital. The procedure of lumbar puncture was performed without problems and patients recovered quickly. Thirty-three of the CSF samples were collected from patients diagnosed with PD and 101 samples were derived from neurologically healthy controls (Table 1). The control patients underwent lumbar puncture after neurological symptoms (e.g., headache, dizziness) to exclude diseases of the central nervous system. Regarding the medication of the PD patients, they are divided into 18 patients without any PD-specific medication, 11 patients with PD-specific medication (one or more of the following drugs: L-dopa, Madopar^®^, Clarium^®^, Sifrol^®^/Pramipexol, Azilect^®^, amantadine, and Artane^®^), and two patients had electrodes for deep brain stimulation.

**TABLE 1:** Characteristics of PD and controls.

	**Parkinson patients**	**Healthy controls**
Number of CSF samples	*n* = 33	*n* = 101
Age average (years)	65.1 ± 12.9	44.8 ± 17.3
Sex (f/m)	10/23	63/38
Duration of disease (years)	1.39 ± 3.7	–

Once lumbar punctuation had been performed, the samples remained at room temperature for no longer than 6 h for routine diagnostics. Subsequently, the samples were temporarily stored at -20 ± 1°C and later at -80 ± 1°C until measurement. The count of erythrocytes was determined in a semi-quantitative manner in a counting chamber (negative = no erythrocytes, isolated < 5 erythrocytes/μl, + < 90 erythrocytes/μl, + + > 90 erythrocytes/μl, + + + > 350 erythrocytes/μl, plentiful = overlying erythrocyte layers). Only samples with negative or isolated erythrocytes were involved in the study. The study was approved by the Ethics Committee of the University Cologne (09.12.2014, no. 14-364). All patients consented to scientific use of their CSF samples.

### Metabolomics: Sample Preparation and Measurement

Prior to FT-ICR-MS analyses, a PPE was performed following the protocol of Willkommen et al. (2018a). The measurement of metabolic features was acquired by means of FT-ICR-MS (Solarix, Bruker, Bremen, Germany), equipped with a 12-T superconducting magnet (Magnex Scientific, Varian Inc., Oxford, United Kingdom) and an ESI source (Apollo II, Bruker Daltonics, Bremen, Germany) as shown in the detailed description in Willkommen et al. (2018a).

### Metallomics: Measurement

The procedure of determination of total concentrations of metals by ICP-sf-MS and the species characterization by SEC-ICP-DRC-MS is reported in Willkommen et al. (2018b).

Since SEC-ICP-DRC-MS measurements and the statistical analysis showed especially the AA fraction to be the most significant influencing factor in the differentiation of PD and control, we decided to additionally separate AAs by the method AAA-direct^TM^. The method is explained in detail in Thermo Scientific (2018). Fractions were collected manually from the AAs Arg, Glu, Gln, Ala, Gly, Ser, Ile, Leu, SeM, His, Phe, and Tyr. These fractions were further analyzed using ICP-sf-MS to determine the elemental concentration of Cu, Fe, Mg, Mn, Ni, Sr, and Zn. The samples were mainly diluted to obtain a ratio of 1:25 (but also 1:100, 1:50, and 1:33 depending on the available sample volume). An eight-point calibration was carried out with concentrations of 10, 50, 100, 250, 500 (ng/L), 1, 5, and 10 (μg/L). Moreover, Rh was added as internal standard and was constantly introduced with a final concentration of 1 μg/L. The instrumental settings of ICP-sf-MS were the following: 1170W RF power, 16 L Ar/min plasma gas flow, 0.99 L Ar/min nebulizer gas flow, and 0.64 L Ar/min auxiliary gas flow. The analytes ^24^Mg, ^25^Mg, ^55^Mn, ^56^Fe, ^60^Ni, ^63^Cu, ^66^Zn, ^87^Sr, and ^103^Rh were measured in medium resolution.

### Data Pre-treatment and Statistics

We integrated the two different datasets with a vertical integration method (Yu and Zeng, 2018). We used the list of most informative metabolites (in total 243 masses, Supplementary Table S2) obtained after applying the feature selection algorithm (Relief) to the entire dataset, as detailed in Willkommen et al. (2018a).

For the metallomic measurements, we used the total concentrations and species characterization of Cu, Fe, Mn, and Zn as well as metal determinations in AA fractions; the data are presented in Willkommen et al. (2018b). The data were vertically integrated together in order to get for each sample two different measurement typologies: metallomic and metabolomics. The dataset was unite variance (UV) scaled and analyzed through a Block-sPLS that represents a suitable tool for data integration (MixOmics package, RStudio Version 1.0.136© 2009–2016 RStudio, Inc.). The model imposed sparseness within the latent components and this is going to improve the variables selection while it performed simultaneous dimension reduction. On the other hand, the block permitted to aggregate two different datasets (Wold, 1966; Tenenhaus, 1998; Tenenhaus and Tenenhaus, 2011). We tested its ability to classify and to discriminate. The classification performance was evaluated by using a sevenfold cross-validation. The balanced error rate (BER) has been calculated to evaluate the model performances. BER is appropriate in case of an unbalanced number of samples per class, as it calculates the average proportion of wrongly classified samples in each class (Rohart et al., 2017). As a result, the model inferred strongly interrelated masses and metallomic variable. By further studying and evaluating lists of correlated variables, we found them to show a particular alteration in PD patients. They were also found to drive the separation between control and PD patients. Additionally, for each of the most high-related metallomic and metabolomics data, we run a general linear model (GLM). The *p* values were calculated with the confounding factors (age and gender) and they were adjusted with the Benjamini–Hochberg test. The experimental design being unbalanced, we estimated the least squares means (LS-means) that correspond to the specified effects for the linear predictor part of the model. The elaborations were done using SAS version 9.3 (SAS Institute Inc., Cary, NC, United States). The data were visualized by different box plots. Moreover, we focused on the reduced list of biomarkers (Supplementary Table S1) already presented in Willkommen et al. (2018a) and we investigated the metallomic features that were most related with the presented m/z values.

## Results and Discussion

**FIGURE 1:**
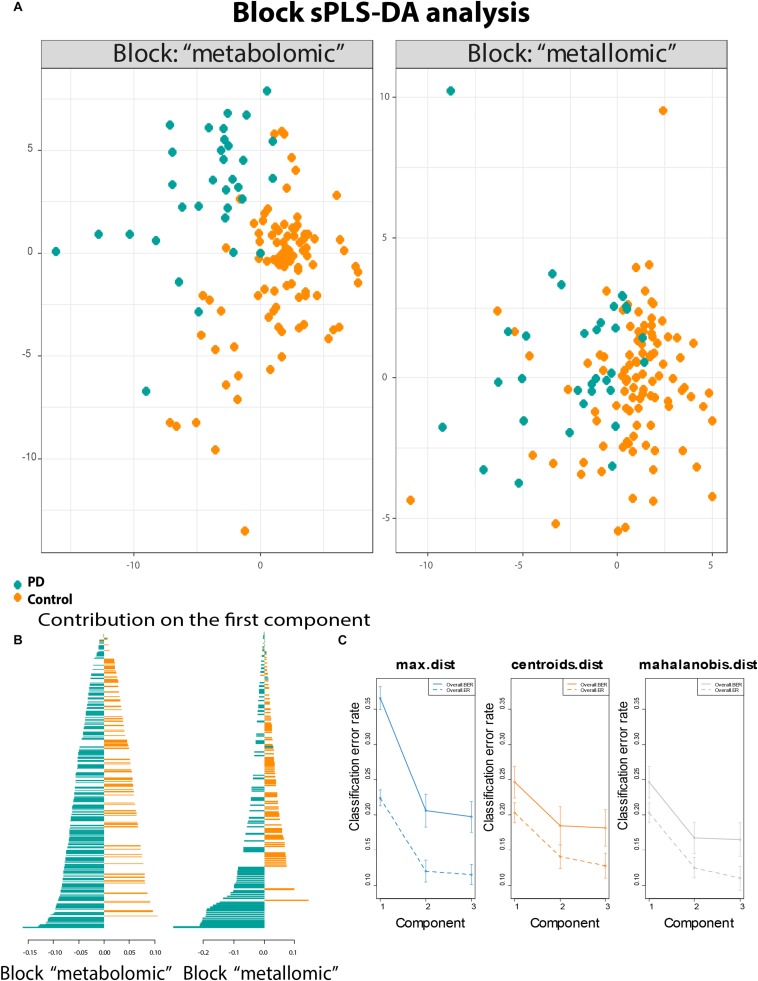
Statistical modeling of the two datasets. **(A)** Map of the samples belonging to the block of metallomics and metabolomics that showed separately the control vs. disease separation; **(B)** the correlation values for the PD patients and Control divided for the metabolomic and metallomic blocks showing increased concentrations/ratios for either PD or Control; and **(C)** classification performance, the error rates stabilized after the second component.

The neurodegenerative disease PD has several factors influencing its onset and progression. Metals, as well as metabolites, show altered concentrations in the human biofluids such as blood, serum, urine, and CSF. In particular, CSF is a suitable biofluid to investigate changes in neurodegenerative diseases. It is in close contact with the brain, and therefore directly connected to the extracellular space of brain parenchyma (Blennow et al., 2010). Therefore, the metabolic changes within the brain are likely to be reflected in CSF (Michalke and Berthele, 2011). The investigations done within the same sample set to obtain the changes in metallomics and metabolomics between PD patients and controls have already been published separately in Willkommen et al. (2018a, b). The present work aimed to correlate the results of both studies to explore new relations between metals and metabolites, thereby opening new hypotheses to explain the mutual relations between different variables. The statistical evaluation of the data could prove the interrelation between the different data (Supplementary Figure S1). On top of the data management, we could confirm a biological agreement between the most correlated variables. The Block sPLS-DA analysis (Figure 1A) visualizes the two main blocks of data (metallomics/metabolomics) and how they could separate the control versus PD patients. The interrelationship between the two initial datasets was found to be at a level of about 47%. Taking into account the different data origin (already statistical reduced) and the different typology of the data, this correlation value is robust/strong enough to allow for further statistical evaluation (illustrated in the Supplementary Figure S1). The analysis has three valid components, absorbing 25% (for the metabolomic dataset) and 23% (for the metallomic dataset) of the total variance. The validity of the component is presented in Figure 1C. The separation between control and PD patients was driven by different variables ranked from the most important to the least important (Figure 1B). The most important variables (with the highest-ranking score) defined the structure of the connection between the most altered variables in the two classes. Mostly the metallomic variables are on the top of such list.

Among the top of the most important variables (Figure 1B), we considered the metallomic variables that were more related to the already published biomarkers. Based on different strengths of the loadings values, which determined the rank, we listed the metallomic and past biomarkers in two different tables (Tables 2, 3). In Table 2, we have listed the 18 variables within the 95th percentile of the most important variables. Table 3 contains the 10 variables within the 50th and 95th percentile of the most important variables. The percentiles were calculated based on the loading values. The listed *p* values were calculated, including, in the models, the covariates (age and gender) to control the demographic influence. Moreover, they were corrected by Benjamini–Hochberg that controls the false discovery rate.

**TABLE 2:** The first group of related variables and their *p* values (test the difference, Control vs. Disease).

**Type of measurement**	**Variables**	**Adjusted *p* value^a^**
Metallomics	IOS (Zn)	0.0146
Metallomics	AA_(Fe)_ vs. AA_(Cu)_	0.0199
Metallomics	Cit_(Zn)_ vs. AA_(Cu)_	0.6381
Metallomics	Cu (HMM%)	0.1081
Metabolomics	p-cresol sulfate	0.0006
Metallomics	IOS_(Zn)_ vs. AA_(Cu)_	0.1781
Metallomics	Fe (IOS)	0.6447
Metallomics	LMM_(Mn)_ vs. LMM_(Cu)_	0.0261
Metallomics	AA_(Mn)_ vs. LMM_(Cu)_	0.3393
Metallomics	Fe (LMM + IOS)	0.7717
Metallomics	IOS_(Zn)_ vs. AA_(Cu)_	0.5994
Metallomics	total c_(Fe)_ vs. LMM_(Cu)_	0.0069
Metallomics	LMM/IOS_(Zn)_ vs. AA_(Cu)_	0.3438
Metallomics	LMM_(Mn)_ vs. AA_(Cu)_	0.0223
Metallomics	IOS_(Fe)_ vs. AA_(Cu)_	0.1049
Metallomics	total c_(Fe)_ vs. AA_(Cu)_	0.0245
Metallomics	IOS_(Mn)_ vs. AA_(Cu)_	0.0278
Metallomics	total c_(Fe)_ vs. AA_(Cu)_	0.1349

*The *p* values are calculated with the confounding factors (age and gender), and they are adjusted with Benjamini–Hochberg. AA, amino acid; c, concentration; Cit, citrate; HMM, high molecular mass; IOS, inorganic species; LMM, low molecular mass. ^*a*^Models with the confounding factors (age and gender).*

**TABLE 3:** The second group of related variables and their *p* values (test the difference, Control vs. Disease).

**Type of measurement**	**Variables**	**Adjusted *p* value^a^**
Metallomics	Ni (Glu) Ni associated with glutamate	0.6634
Metallomics	Ni (Ile)	0.3033
Metabolomics	Valerenic acid	0.1180
Metabolomics	Mannosylglycerate	0.4745
Metabolomics	Quinic acid	0.0291
Metallomics	Cu (His) Cu associated with histidine	<0.0001
Metabolomics	Arachidonic acid	0.0013
Metabolomics	Decanoic acid	0.7490
Metallomics	Ni (Arg) Ni associated with arginine	0.1524
Metabolomics	10-Hydroxydecanoic acid	0.0125

*The *p* values are calculated with the confounding factors (age and gender) and they are adjusted with Benjamini–Hochberg. ^*a*^Models with the confounding factors (age and gender).*

**FIGURE 2:**
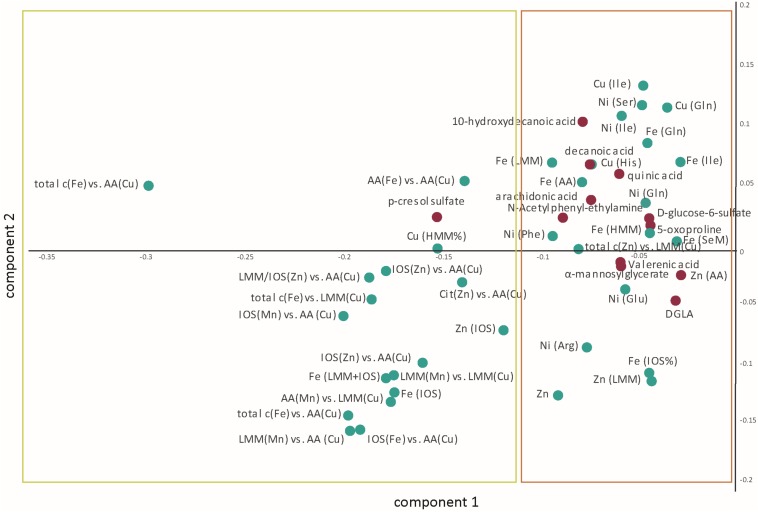
The resulting loadings plot structure (in the first and second component) aimed at highlighting the multiple interrelations between the metallomic (green dots) and the previously defined biomarkers (red dots). The variables are up-regulated in PD. The yellow box contains the variables presented in Table 2, while the orange box contains the variables listed in Table 3. AA, amino acid; Arg, arginine; Cit, citrate; DGLA, dihomo-γ-linoleic acid; Gln, glutamine; Glu, glutamic acid; His, histidine; HMM, high molecular mass; Ile, isoleucine; IOS, inorganic species; LMM, low molecular mass; Phe, phenylalanine; SeM, selenomethionine; Ser, serine.

**FIGURE 3:**
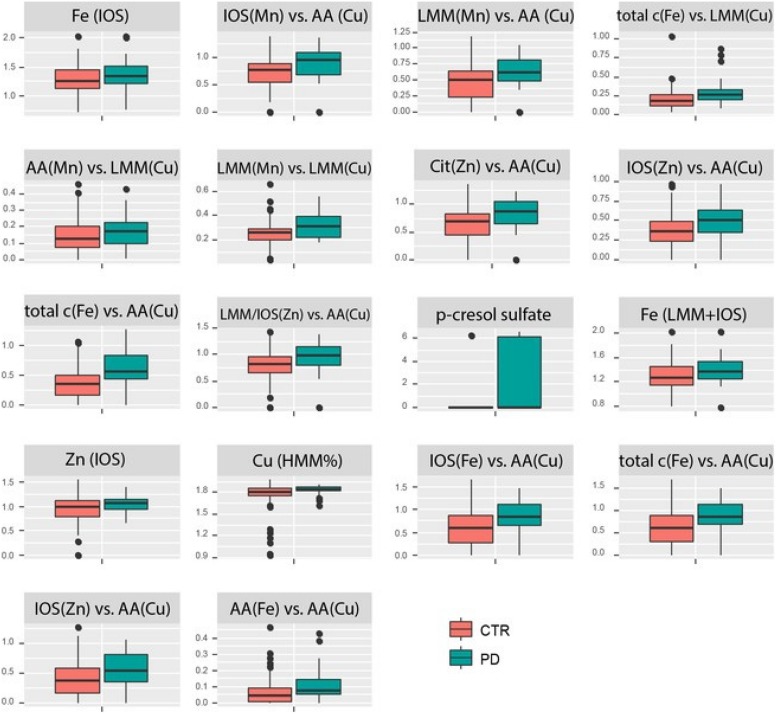
Box plots of the individual variations in PD compared to control for the strongest correlating parameters. All concentrations/ratios are increased in PD.

Within the 95th percentile of the most important variables (yellow box in Figure 2), mainly metallomic parameters are involved. The first block (Table 2) of strong positive correlations arose among the significantly differentiating ratios of size fractions. Additionally, specific fractions of the SEC analysis of Fe, Cu, Mn, and Zn were involved in these correlations as well. For the strongest relations, we presented also the box plots for individual variation (Figure 3). All parameters show an increased loadings value in PD patients. The close linkage among the variables is attributable to the involvement of LMM fractions of Cu within the ratios or more specifically the AA fractions of Cu as described in Willkommen et al. (2018b). Figure 2 shows that all the 13 ratios with a Cu fraction in the denominator are increased in PD patients. Moreover, the important role of the redox-active trace element Fe is underlined. This element is also found within these highly significant correlations. The size fractions of Fe, as well as all ratios having Fe in the numerator show increased concentration/rate in PD patients, as illustrated in the respective box plots in Figure 3. Both trace elements (Cu, Fe) evolve their toxicity through the ability to form ROS via Fenton reaction. Within the cascade of oxidative reactions hydroxyl radicals are synthesized leading to lipid peroxidation. Moreover, metallo-enzymes with Cu or Fe in the active center may contribute to altered redox balance and are known to contribute to the neuropathology of diseases like PD. Additionally, Cu as well as Fe are involved in the abnormal protein aggregation which is a major hallmark of neurodegenerative diseases (Gaeta and Hider, 2009). The dyshomeostasis of various elemental balances seems to be impaired in PD and may be an attributor and major influence on disease development. The strict regulation of metal ratios was already established to preserve proper brain function (Ahmed and Santosh, 2010; Zheng and Monnot, 2012; Zhao et al., 2013). Additionally, this block of metallomic parameters shows a strong positive correlation with the metabolite p-cresol sulfate in PD CSF samples. The compound p-cresol sulfate is linked to the neurological disease multiple sclerosis (Cao et al., 2000). Moreover, this metabolite is a metabolic waste product that can be found in CSF (Cassol et al., 2014). Metabolic waste products, like p-cresol sulfate, show increased accumulation also in Alzheimer’s disease and, therefore, are a hallmark of several age-associated neurodegenerative diseases (Nedergaard, 2013). Our results also indicated a marked increase in the concentration of p-cresol sulfate in CSF (box plot in Figure 3). The compound p-cresol sulfate is a breakdown product of Tyr and phenylalanine and therefore also closely connected with the AAs and, by extension, the AA fractions and their ratios. Thus, the close connection of this metabolic waste product with the AA size fractions of the species characterization supports the effectiveness of our analyses. The increase of metabolic waste is paralleled by an increase of AAs in CSF.

Apart from the ratios of various elemental balances, in the second block, we found various correlations between the fatty acid metabolites arachidonic acid, decanoic acid, 10-hydroxydecanoic acid, valerenic acid, and quinic acid with various metals analyzed within the AA fractions. A list of these correlating parameters is gathered in Table 3. The fatty acids are highly correlated through the lipid metabolism within human biology. In addition, correlations to the redox-active metals Cu and Fe are involved. As already mentioned, both metals are essential trace elements in the human body. But if they are in excess, they can be deleterious to the metabolism through ROS (Gaeta and Hider, 2009). The fatty acid decanoic acid was found with increased concentration within the PD-CSF samples in our study (Willkommen et al., 2018a). Hughes et al. (2014) exposed neuronal cells with decanoic acid and identified an increased catalase activity after 6 days of exposure. Catalase is an antioxidant compound removing the hydrogen peroxide produced by SOD (Scibior and Czeczot, 2006). In humans, three forms of SOD are present, including the Cu-Zn-SOD. This latter SOD is a metallo-enzyme, which is known to protect against oxidative stress. The increased Cu associated with histidine might be a part of this SOD metallo-enzyme and it is positively correlated with increased concentration of decanoic acid. Both compounds are metabolically linked closely to each other and they can hint increased oxidative stress conditions in PD patients. Increased oxidative stress is a intensively discussed influencing parameter in neurodegenerative diseases (Andersen, 2004; Kim et al., 2015; Liu et al., 2017). In addition to Cu and Fe, the essential trace element Ni is associated with the fatty acids. Ni was measured in the manually taken fractions of AA analysis and seems to be associated with them. The metal Ni is redox active, as are Fe and Cu, and it is part of several metallo-enzymes in which it acts as the catalytic center, e.g., in SOD (Ryan et al., 2015) and acetyl-CoA-synthase (Boer et al., 2014; Can et al., 2014; Manesis et al., 2017). In this context, the positive relations between fatty acids and Ni found within AAs fractions is of great importance, since the metallo-enzymes acetyl-CoA-synthase is necessary for the production of acetyl-CoA, which in turn is a major brick in fatty acid synthesis (Ikeda et al., 2001). Therefore, the positive correlation between increasing activity of acetyl-CoA-synthase with Ni in its catalytic center to produce acetyl-CoA along with the increasing concentrations of fatty acids seems to be closely related. This connection needs further investigations to uncover the real influence of Ni in PD. Especially, the influence of Ni in connection to acetyl-CoA-synthase and fatty acid synthesis seems to be a promising leverage point. Ni was already investigated in various biofluids of PD patients in comparison to controls. Maass et al. (2018) determined an elemental fingerprint in CSF samples. The results could not identify a significant difference in the Ni level, but ascertained the metal among others for sufficient classification between PD and control (Maass et al., 2018). Furthermore, a study investigating various metals in serum and plasma showed significant increased Ni concentrations in serum as well as in blood (Forte et al., 2005). Both studies showed increased Ni concentrations in PD identifying the trace element as a possibly important contributor to PD progression, which is in agreement with our findings.

The data integration of complementary Omics approaches, namely, metallomics and metabolomics, was done to enhance knowledge of potential causative changes associated with PD. Sophisticated statistical models were optimized and applied to prove the correlations and uncover alternative influencing factors. We identified two groups of correlating variables highlighting the impact of metallomic as well as metabolomic parameters to disease development. The integrative metabolomic and metallomic analysis showed the importance of such models. Connections between parameters of various types of analyses are revealed and give a broader overview. In this research, our data integration opens new perspectives and shows once more its important impact on data interpretation and on disclosing new information about biological phenomena that would go unnoticed otherwise.

## Data Availability Statement

Publicly available datasets were analyzed in this study. This data can be found here: Willkommen et al. (2018a, b).

## Ethics Statement

The studies involving human participants were reviewed and approved by the Ethics Committee of the University of Cologne (09.12.2014, no. 14–364). All patients consented to the scientific use of their CSF samples. The patients/participants provided their written informed consent to participate in this study.

## Author Contributions

DW, ML, PS-K, and BM conceived the study and developed the methodology. ML contributed to the data curation, formal analysis, and visualization. DW and ML investigated and validated the results and wrote the original draft. DW, MS, PS-K, and BM administered the project. MS, AS, BM, and PS-K provided the resources. PS-K and BM supervised the study. DW, ML, MS, AS, PS-K, and BM reviewed and edited the manuscript.

## Conflict of Interest

MS received personal compensation for talks and advisory boards by Biogen, Sanofi/Genzyme, Grifols, Merck, Miltenyi Biotec, Novartis, and Roche. The remaining authors declare that the research was conducted in the absence of any commercial or financial relationships that could be construed as a potential conflict of interest.

## Abbreviations

AA: amino acids
Ala: alanine
Arg: arginine
Block-sPLS-DA: Blocks Sparse Partial Least Square Discriminant Analysis
Cit: citrate
DGLA: dihomo- γ-linolenic acid
DRC: dynamic reaction cell
ESI: electrospray ionization
FT-ICR-MS: Fourier transform-ion cyclotron resonance-mass spectrometry
Gln: glutamine
Glu: glutamic acid
Gly: glycine
His: histidine
HMM: high molecular mass
ICP-MS: inductively coupled plasma-mass spectrometry
Ile: isoleucine
IOS: inorganic species
Leu: leucine
LMM: low molecular mass
m/z: mass-to-charge ratio
PD: Parkinson’s disease
Phe: phenylalanine
PPE: protein precipitation extraction
ROS: reactive oxygen species
SEC: size exclusion chromatography
SeM: selenomethionine
Ser: serine
sf: sector field
SOD: superoxide dismutase
Tyr: tyrosine
