# Supplementary material for: Integrative Metabolomic and Metallomic Analysis in a Case–Control Cohort With Parkinson’s Disease
Source: Front Aging Neurosci. 2019 Dec 6;11:331. doi: 10.3389/fnagi.2019.00331 (PMC6908950; doi:10.3389/fnagi.2019.00331)

Integrative Metabolomic and metallomic analysis in a case control cohort with Parkinson´s disease

Authors: Marianna Lucio[1*¶], Desiree Willkommen[1¶], Michael Schroeter[2], Ali Sigaroudi[3,4], Philippe Schmitt-Kopplin[1,5], Bernhard Michalke[1]

[1] Analytische BioGeoChemie, Helmholtz Zentrum München, Neuherberg, Germany

[2] Uniklinik Köln, Klinik und Poliklinik für Neurologie, Kerpener Str. 62; 50937 Köln

[3] Universitätsspital Zürich, Klinik für Klinische Pharmakologie und Toxikologie, Rämistr. 100, 8091 Zürich

[4] Uniklinik Köln, Institut I für Pharmakologie, Zentrum für Pharmakologie, Gleueler Str. 24, 50931 Köln

[5] TU Munich, Chair of analytical food chemistry, Science center Weihenstephan, Freising, Germany

¶Equally contributed

* Corresponding author:
Marianna Lucio

marianna.lucio@helmholtz-muenchen.de

Keywords: Data integration, Metabolomics, Metallomics, Block-sPLS-DA, Parkinson´s disease.

Supporting Information

Table S1: Assigned and significantly differentiating compounds of metabolomics investigation.

Table S2: List of metabolites obtained after the feature selection algorithm, published in (Willkommen *et al.*, 2018*a*)

Figure S1: Illustration of correlation between metallomic and metabolomic data out of the same sample set with respective correlation value.

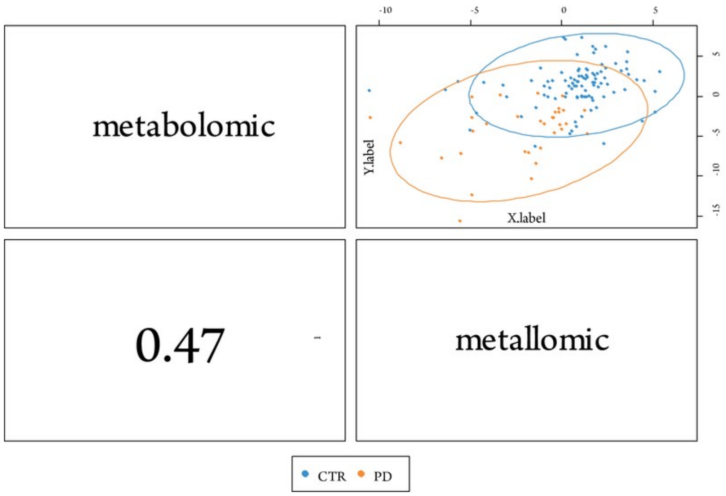

Supplement: Supplementary file 3 [file Data_Sheet_1.pdf]
